# Versatile Applications of Silver Nanowire-Based Electrodes and Their Impacts

**DOI:** 10.3390/mi14030562

**Published:** 2023-02-27

**Authors:** Chunghyeon Choi, Erik Schlenker, Heebo Ha, Jun Young Cheong, Byungil Hwang

**Affiliations:** 1 School of Integrative Engineering, Chung-Ang University, Seoul 06974, Republic of Korea; 2 College of Health, Science and Technology at University of Illinois Springfield, One University Plaza, Springfield, IL 62703, USA; 3 Bavarian Center for Battery Technology (BayBatt) and Department of Chemistry, University of Bayreuth, Universitätsstraße 30, 95447 Bayreuth, Germany

**Keywords:** silver, nanowire, synthesis, application, electrode

## Abstract

Indium tin oxide (ITO) is currently the most widely used material for transparent electrodes; however, it has several drawbacks, including high cost, brittleness, and environmental concerns. Silver nanowires (AgNWs) are promising alternatives to ITO as materials for transparent electrodes owing to their high electrical conductivity, transparency in the visible range of wavelengths, and flexibility. AgNWs are effective for various electronic device applications, such as touch panels, biosensors, and solar cells. However, the high synthesis cost of AgNWs and their poor stability to external chemical and mechanical damages are significant challenges that need to be addressed. In this review paper, we discuss the current state of research on AgNW transparent electrodes, including their synthesis, properties, and potential applications.

## 1. Introduction

Transparent electrodes are crucial components in various electronic devices such as displays [1,2], solar cells [3,4], and energy harvesters [5,6]. Indium tin oxide (ITO) shows high optical transmittance and electrical conductivity, as well as excellent chemical stability, which makes it a leading material system for the transparent electrodes of various electronic devices. However, ITO is intrinsically brittle under harsh deformation, which limits its use for flexible/wearable devices [7,8]. Furthermore, ITO needs the post-annealing process under high temperatures to enhance electrical conductivity, which is not suitable for polymer-based flexible substrates. The cost of indium is also relatively high [7,8]. Hence, in recent years, there has been significant research interest in developing alternative transparent electrode materials. One promising candidate is silver nanowires (AgNWs) [9,10,11]. In this review paper, we discuss the current state of research on AgNW transparent electrodes, including their synthesis, properties, and potential applications.

**Figure 1:**
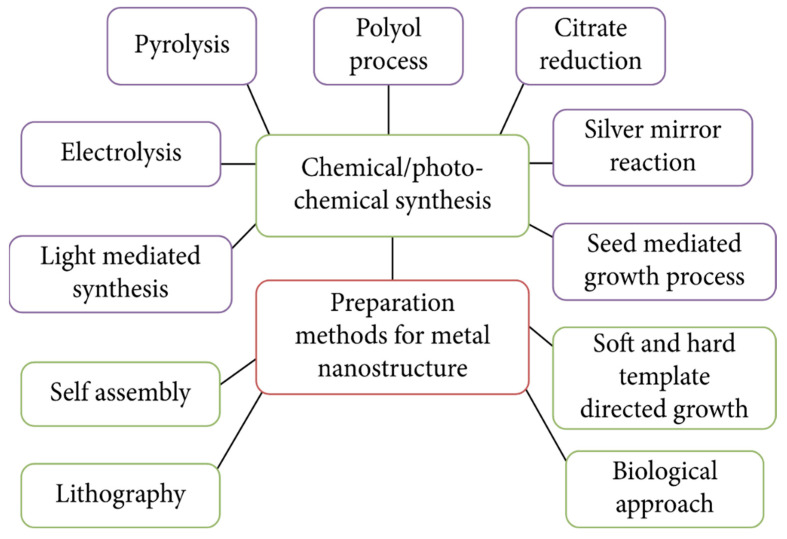
Different methods of synthesis of metal nanostructures. Reproduced from Ref. [26] under the Creative Commons Attribution 4.0 International (CC BY 4.0) License.

AgNWs are cylindrical in shape with diameters in the order of tens of nanometers and lengths in the order of micrometers [12,13]. AgNWs can be synthesized through various methods, including chemical synthesis [14,15], electrodeposition [16,17], and physical vapor deposition [18]. Chemical synthesis methods, such as the polyol reduction process, are effective in producing high-quality AgNWs with high aspect ratios [14,15]. The electrical and optical properties of AgNW transparent electrodes are significantly dependent on the aspect ratio of individual AgNWs and the density of the deposited networks [14,15]. To achieve high electrical conductivity with optical transparency, long and thin AgNWs are required because they can make sufficiently percolated networks with a low density of AgNWs that provide sufficient vacant spaces through which light can transmit without a severe reflection at the surface of AgNWs [14,15]. However, AgNWs have several drawbacks that need to be addressed. One of the main challenges is the high synthesis cost of AgNWs and the lack of scalability of the process. Additionally, AgNWs have poor stability to external chemical and mechanical damages [19,20]. To overcome these limitations, various hybrid systems have been proposed such as AgNW metal oxide [11,20,21,22], AgNW polymer [7,23], and AgNW graphene [24,25]. Each hybrid system has certain pros and cons; thus, appropriate integration of different materials systems is required depending on the application. In this review, different material systems used for different applications (Figure 1) are discussed.

## 2. Synthesis of AgNWs

The synthesis of AgNWs is challenging owing to the difficulty of controlling their size, shape, and morphology. In this review paper, we discuss various synthetic methods of AgNWs, their pros and cons, and the detailed procedures and materials used in each method.

### 2.1. Chemical Reduction Method

**Figure 2:**
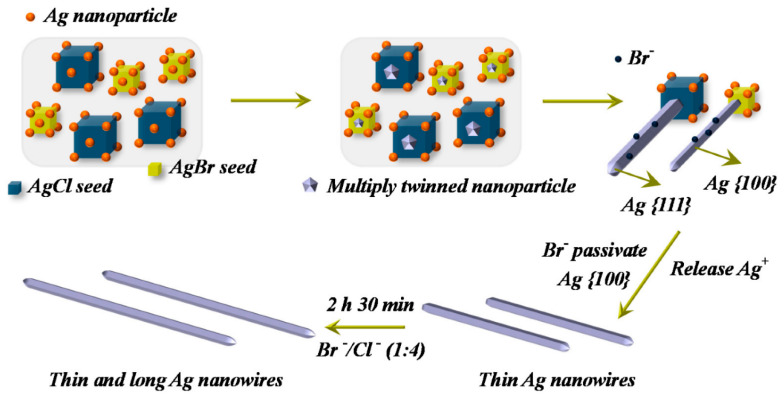
Growth mechanism of AgNWs via chemical reduction process. Reproduced from Ref. [27] under the Creative Commons Attribution 4.0 International (CC BY 4.0) License.

The chemical reduction method is one of the most widely used ways to synthesize AgNWs [14,15,27]. Silver ions are reduced in a solution such as water or ethanol using a reducing agent such as sodium borohydride or hydrazine (Figure 2) [27]. The mixture is then heated to ~90 °C, for 1~3 h. The synthesized AgNWs are then purified and collected through centrifugation or filtration.

The advantages of the chemical reduction method include its simplicity, low cost, and the quality of the produced AgNWs with a high aspect ratio [14,15]. However, the difficulty in controlling the size and shape of the synthesized AgNWs and the contamination with impurities limit their use in the industry [14,15]. In addition, the chemical reduction method often leads to the formation of Ag nanoparticles (AgNPs) along with AgNWs, which can cause a hazard issue [14,15].

### 2.2. Template-Assisted Method

**Figure 3:**
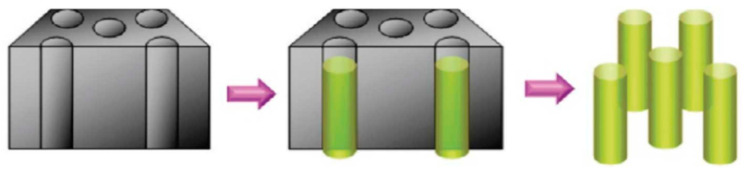
A schematic illustration of the growth process of nanowires via a hard template. Reproduced from Ref. [26] under the Creative Commons Attribution 4.0 International (CC BY 4.0) License.

Another method for the synthesis of AgNWs is the template-assisted method [26,28]. This method involves the use of a template, such as a porous anodized alumina (PAA) template or a polycarbonate (PC) template, to guide the growth of AgNWs (Figure 3) [26]. The procedure involves coating the template with a thin layer of AgNWs using a chemical reduction method or a physical vapor deposition method, followed by the removal of the template by etching or dissolution [29].

The template-assisted method allows control over the size and shape of the AgNWs and yields AgNWs with a high aspect ratio [29]. However, the disadvantages of this method include the high cost of the template materials and the difficulty of removing the template from the AgNWs after the growth process [30]. Additionally, the template-assisted method may lead to the formation of AgNPs along with AgNWs, which can prevent one from solely investigating the role of AgNWs.

### 2.3. Template-Assisted Electrodeposition Method

**Figure 4:**
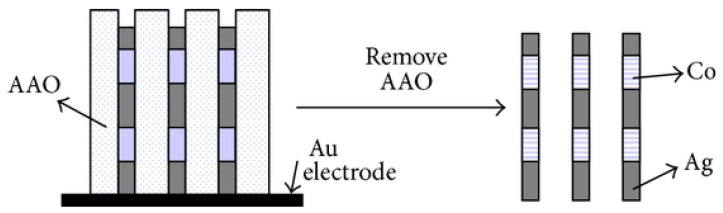
The synthesis process of the mechanism of nanowires via PAA templates. Reproduced from Ref. [31] under the Creative Commons Attribution 4.0 International (CC BY 4.0) License.

The third method for the synthesis of AgNWs is the electrodeposition method [31,32]. This method involves the electrodeposition of AgNWs on an electrode surface using an electric current. An electrode is immersed in a solution containing silver ions and a supporting electrolyte, and an electric current is applied to the electrode that creates AgNWs (Figure 4). The AgNWs are then collected by removing the electrode from the solution. The electrodeposition method can also control the size and shape of the AgNWs by adjusting the conditions of electrodeposition [31]. However, the surface of the AgNWs synthesized by electrodeposition is rough, which can lead to light reflection. In addition, mass production is also limited.

## 3. Applications of AgNWs

### 3.1. Touch Screens

**Figure 5:**
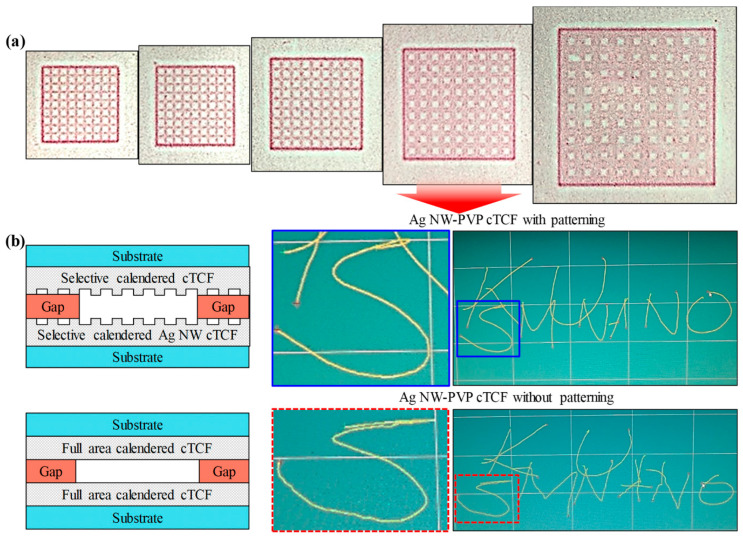
Resistive touch sensing panels fabricated by selective calendering of Ag NW-PVP cTCFs: (**a**) a mesh pattern imaged on pressure-sensitive paper with various line widths, (**b**) a cross-section structure and performance of touch sensing panel between selectively calendered (upper) and full area calendered (bottom) Ag NW-PVP TCF with a mesh pattern. Reproduced from Ref. [33] under the Creative Commons Attribution 4.0 International (CC BY 4.0) License.

AgNW transparent electrodes have high optical transmittance and electrical conductivity, which make them suitable for use in touch screens for electronic devices (Figure 5) [33,34]. Touch panels using AgNW electrodes utilize AgNWs as transparent electrodes. Different types of electrodes have been used for AgNW-based touch screens [35]. The AgNW network is a simple system where only AgNWs are used to form transparent electrodes. The touch panel works by measuring the change in electrical resistance at the point of contact. However, the limited chemical stability of the bare AgNW networks restricts their use in the industry. To overcome the stability issues, AgNW-ITO hybrid types are proposed [36]. ITO provides increased electrical conductivity as well as enhanced chemical stability. However, the brittleness of ITO degrades the mechanical flexibility of the AgNW networks. Another strategy is using AgNW-printed electrodes [37]. AgNW ink formulated for enhanced chemical resistance is printed on flexible substrates. The stability of AgNWs can be tuned by the type of ink formulation. However, the cost of the ink formulation is high and the minimum pattern size is micron-scale; these factors limit their use for state-of-the-art miniaturized devices. AgNW-embedded electrodes have high chemical and mechanical stabilities while maintaining the excellent optical and electrical properties of AgNWs [7]. AgNWs are embedded in the surface of the flexible polymeric matrix, which protects AgNWs from external mechanical and chemical damage [7]. However, the embedding process is complex, and it can reduce the mass production of AgNW electrodes. The specific technology depends on the requirements of the application, including the level of transparency and electrical conductivity required, as well as the cost and durability of the touch panel.

### 3.2. Solar Cells

**Figure 6:**
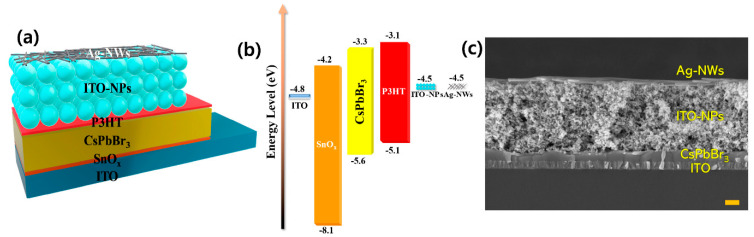
(**a**) Schematic illustration of an AgNW solar cell: constructed n–i–p planar CsPbBr_3_ perovskite solar cell architecture, (**b**) designed energy band diagram, and (**c**) SEM image of the cross-sectional device (scale bar = 300 nm). Reproduced from Ref. [47] under the Creative Commons Attribution 4.0 International (CC BY 4.0) License.

There have been many reports on using AgNW electrodes in solar cells [38]. The reports showed that AgNW electrodes have a high power conversion efficiency, which is attributed to their high transparency and electrical conductivity [39,40]. This makes them suitable for use as transparent electrodes in solar cells. Several different technologies can be used to create solar cells using AgNW electrodes, including dye-sensitized solar cells (DSSCs) [41,42], organic solar cells (OSCs) [43,44], and hybrid perovskite solar cells (HPSCs) [45,46]. These solar cells work by absorbing light with photovoltaic materials, which leads to the formation of an electron hole pair. The AgNW electrode is used to collect the electrons and transfer them to an external circuit. All these technologies have certain advantages and limitations. Dye-sensitized solar cells (DSSCs) have high efficiency but relatively low stability. Organic solar cells (OSCs) have relatively high stability but low efficiency. Hybrid perovskite solar cells (HPSCs) have high efficiency and stability (Figure 6), but further research is ongoing. The specific technology used depends on the requirements of the application, including the efficiency, stability, and cost of the solar cell.

### 3.3. Transparent Heaters

**Figure 7:**
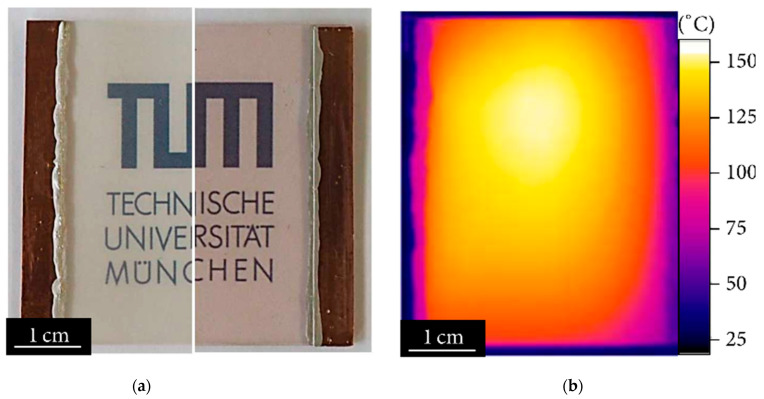
Optical microscope image of an AgNW-based transparent heater. (**a**) shows the highly transparent AgNW layer on the glass substrate; (**b**) the uniform and efficient heating performance. Reproduced from Ref. [51] under the Creative Commons Attribution 4.0 International (CC BY 4.0) License.

AgNWs have high thermal conductivity and can be used as transparent heaters in various electronic devices, such as smartphones and tablets, as well as in automotive and building applications [48,49]. Transparent heaters require high optical transparency to be used for various transparent substrates, such as glass windows or transparent heating films [48,49,50]. In addition, high electrical conductivity is also important to achieve a uniform and efficient heating performance. Thus, AgNWs with a high optical transmittance and electrical conductivity are attractive for the conductive layer of the transparent heater. For example, Bobinger et al. formed the highly transparent AgNW layer on the glass substrate, as shown in Figure 7a, which resulted in a uniform and efficient heating performance (Figure 7b) [51]. Several different technologies can be used to create transparent heaters using AgNW electrodes [48,49,52]. Transparent resistive heater technology involves the use of AgNWs as transparent electrodes in the resistive heating element [48,49]. An electrical current is passed through the AgNWs, causing them to resist the flow of electricity and generate heat. Transparent near-infrared (NIR) heaters involve the use of AgNWs as transparent electrodes in NIR heaters [52]. AgNWs are used to absorb and convert NIR light into heat. In transparent microwave heaters, AgNWs are used to convert microwave energy into heat [53]. All these technologies have certain advantages and limitations. Transparent joule heating gives a high temperature but relatively low efficiency [48,49]. Transparent NIR heaters have high efficiency but relatively low temperature [52]. Transparent microwave heaters have a high temperature but relatively low efficiency [53]. The specific technology used depends on the requirements of the application, including the temperature, efficiency, safety, and cost of the transparent heater. AgNWs have the advantage of being highly transparent and conductive, making them well-suited for use in transparent heaters.

### 3.4. Anti-Counterfeiting Materials

**Figure 8:**
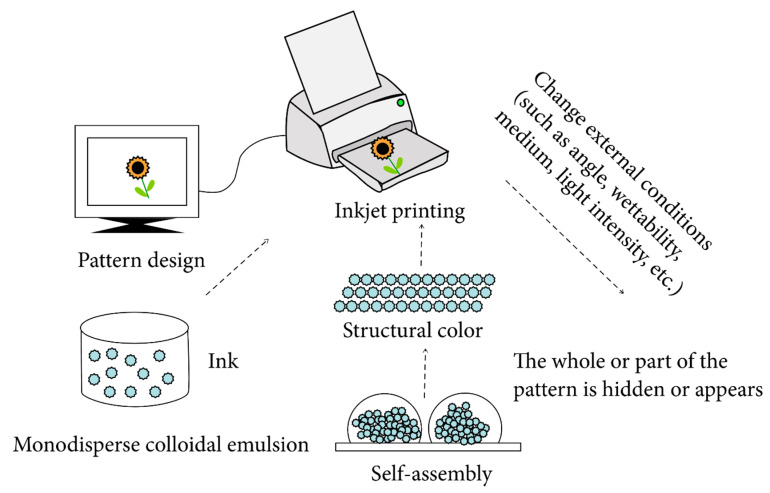
A schematic of the fabrication mechanism of anti-counterfeiting labels using AgNWs. Reproduced from Ref. [54] under the Creative Commons Attribution 4.0 International (CC BY 4.0) License.

AgNWs have unique optical and electrical properties, which can be used to create anti-counterfeiting labels and tags that are difficult to be replicated [54,55]. Anti-counterfeiting technologies use AgNW electrodes as a key component in the design of security features for products (Figure 8). AgNWs are highly conductive and can be used to create transparent electrodes with high conductivity, which can be used in several anti-counterfeiting applications. One example of an anti-counterfeiting technology that uses AgNW electrodes is the creation of transparent security tags [54,55]. These tags use AgNW electrodes to create unique electrical signatures, which can be used to authenticate the product. The electrical signature can be read by a handheld device, such as a smartphone, to verify the authenticity of the product. Another example of an anti-counterfeiting technology using AgNW electrodes is the creation of holographic images, which can be incorporated into packaging or labels. These images use AgNW electrodes to create a unique pattern of light and dark areas, which can be used to authenticate the product.

### 3.5. Biomedical Applications

**Figure 9:**
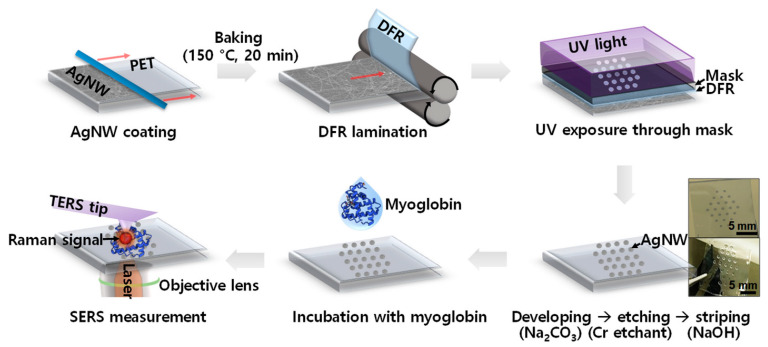
The fabrication of optical biosensors using AgNWs, which is marked by AgNW coating, lamination, UV exposure, etching, striping, incubation, and final measurement. Reproduced from Ref. [56] under the Creative Commons Attribution 4.0 International (CC BY 4.0) License.

AgNWs have excellent biocompatibility and can be used in various biosensors [16,56,57]. In electrochemical biosensors, AgNWs are used to create the working electrode and the reference electrode. The biological molecules are immobilized on the working electrode (AgNWs), and the changes in the electrochemical activity of the biological molecules are detected and measured. Optical biosensors utilize the unique plasmonic resonance that occurs on the surface of AgNWs [56,57]. The AgNWs detect the changes in the optical properties of the biological molecules (Figure 9). The field-effect biosensor utilizes AgNWs as the gate electrode in the biosensor [16]. The biological molecules are immobilized on the gate electrode, and the changes in the electrical properties of the biological molecules are detected and measured. All these technologies have certain advantages and limitations. Electrochemical biosensors have high sensitivity but relatively low selectivity. Optical biosensors have high selectivity but relatively low sensitivity. Field-effect biosensors have high sensitivity and selectivity but have a relatively high cost.

### 3.6. Triboelectric Nanogenerators

**Figure 10:**
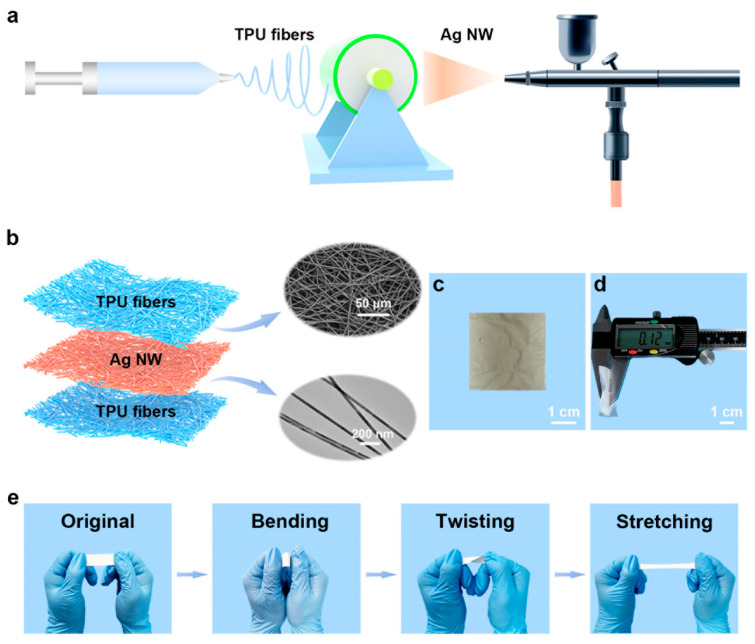
The fabrication and characterization of the TE-skin. (**a**) A schematic diagram of the experimental process for fabricating the TE-skin. (**b**) The sandwich structure of the TE-skin. (**c**) Photograph of the Ag NW electrode layer that is deposited on the TPU fiber substrate. (**d**) Photograph of the TE-skin with a total thickness of 120 µm. (**e**) Photographs of the TE-skin in the original, bent, twisted, and stretched states. Reproduced from Ref. [60] under the Creative Commons Attribution 4.0 International (CC BY 4.0) License.

Triboelectric nanogenerators (TENGs) convert mechanical energy into electrical energy [58,59,60]. They consist of two different materials—one positively charged and the other negatively charged (Figure 10b). When these materials come in contact and are separated, an electrical charge is generated. This principle can be used to convert various types of mechanical energy, such as vibration or pressure, into electrical energy.

Recently, TENGs using AgNW electrodes have been researched as promising technologies for harvesting energy from various sources (Figure 10) [58,59,60]. AgNWs are effective for TENG electrodes owing to their high electrical conductivity and high mechanical strength. Additionally, AgNWs have high flexibility, which is critical for TENGs that need to be flexible to convert mechanical energy into electrical energy. Several techniques have been proposed for fabricating AgNW TENGs, which include using a substrate coated with AgNWs as one of the electrodes in a TENG, using AgNWs as the active material in a TENG by growing AgNWs directly on the triboelectric material, or coating AgNWs on the triboelectric material (Figure 10a), which can enhance the performance of the TENG by increasing the contact area between the triboelectric material and the AgNWs. AgNW TENGs have a high output power density, which is a measure of the amount of electrical power that can be generated per unit area. Additionally, AgNW TENGs have a high energy conversion efficiency, which is a measure of the amount of mechanical energy that can be converted into electrical energy.

## 4. Conclusions

AgNWs are a promising alternative to traditional materials for transparent electrodes. The large aspect ratio of AgNWs results in high electrical conductivity and high optical transmittance, which allowed them to be used for various electronic or energy devices. Several synthesis methods have been reported for AgNWs, including chemical reduction or template-assisted methods. Although each method has specific advantages, the high cost of synthesis and limited mass producibility is required to be resolved in the future. The most critical problem of using AgNWs for the transparent electrode is their poor stability to external chemical or mechanical damage. To overcome these limitations, various hybrid systems have been proposed, such as AgNW metal oxide, AgNW polymer, and AgNW graphene. Each hybrid system needs to be appropriately integrated based on the applications, and further research is needed to address the challenges in the production and application of AgNW transparent electrodes in a simple and scalable manner. In addition, there is still room for AgNWs in various application areas, such as electrocatalysts or electromagnetic shielding. Ag nanowires can produce high-efficiency electrochemical hydrogen production and CO_2_ reduction, as well as electromagnetic shielding, due to their large surface area and excellent electrical conductivity. Such new application areas are required to be studied further.

## Data Availability

The datasets used and/or analyzed during the current study are available from the corresponding author upon reasonable request.
